# Microinvasive Glaucoma Surgery

**DOI:** 10.1155/2017/9845018

**Published:** 2017-06-05

**Authors:** Chelvin C. A. Sng, Paul Harasymowycz, Keith Barton

**Affiliations:** ^1^Department of Ophthalmology, National University Hospital, Singapore; ^2^Glaucoma Service, Moorfields Eye Hospital, London, UK; ^3^Singapore Eye Research Institute, Singapore; ^4^Glaucoma Research Unit, University of Montreal, Montreal, Canada; ^5^Montreal Glaucoma Institute, Montreal, Canada; ^6^National Institute for Health Research, Biomedical Research Centre for Ophthalmology, Moorfields Eye Hospital, London, UK; ^7^Department of Epidemiology and Genetics, Institute of Ophthalmology, University College, London, UK

Microinvasive glaucoma surgery (MIGS) devices have transitioned into mainstream use in recent years. Several devices, including the iStent trabecular microbypass stent, the Cypass microstent and the XEN-45 implant, have obtained the United States Food and Drug Administration (US-FDA) approval in addition to the Conformité Européene Mark. The high safety profile of MIGS allows it to be used earlier than conventional glaucoma surgeries within the glaucoma treatment algorithm, and it is typically combined with cataract surgery in patients with mild-to-moderate primary open-angle glaucoma (POAG).

Long-term data on the efficacy and safety of MIGS devices are limited. In this special issue, P. Arriola-Villalobos reported the first study on the iStent Inject with more than 12 months follow-up. Similar to previous studies on the iStent trabecular microbypass stent, the efficacy of the iStent Inject is modest, with only 28% of patients achieving IOP ≤ 18 mmHg without postoperative medications at 5 years. Though there are two stents in each injector, postoperative gonioscopy revealed only one functional stent in 35% of the patients. This is because the iStent Inject can be “over-implanted” and completely embedded within trabecular tissue, with no contact with aqueous in the anterior chamber. Even if the proximal end of the device is visible in the angle, “under-implantation” is still possible as the distal end may not extend to the Schlemm's canal. Incorrect placement of trabecular bypass devices might be more prevalent than anticipated and may partially explain why they have been termed “minimally effective glaucoma surgery.” Hopefully, newer versions of these devices would allow consistent access to the Schlemm's canal.

This special issue on MIGS has highlighted that these devices are slowly making inroads into Asian countries, pioneered again by the iStent trabecular microbypass stent. The US-FDA has approved the iStent trabecular microbypass stent for use only in conjunction with cataract surgery, and the foray of this device outside USA has allowed it to be “phaco-minus.” D. Shiba et al. reported the first results of two iStent trabecular microbypass stents as a solo procedure in Japanese patients with medically uncontrolled POAG. The 6-month results are similarly modest, possibly attributable to reasons mentioned above, with a 23.2% reduction in IOP on the same number of glaucoma medications. With the globalization of MIGS, the use of these devices is likely to extend into other subtypes of glaucoma, including angle closure glaucoma [1] and uveitic glaucoma [2].

Microinvasive glaucoma surgery has largely been confined to eyes with mild to moderate glaucoma and moderately elevated IOP. However, in experienced hands, it can potentially achieve more remarkable efficacy in eyes with markedly raised IOP. In this special issue, H. Akil et al. reported impressive results with the Trabectome in patients with preoperative IOP of 30 mmHg or higher. The IOP was reduced from 35.6 ± 6.3 mmHg at baseline to 16.8 ± 3.8 mmHg at 12 months, with a reduction in medications from 3.1 ± 1.3 to 1.8 ± 1.4. It is likely that the Schlemm's canal is more consistently assessed when trabecular bypass procedures are performed by experienced surgeons. Hopefully, advancements in ocular imaging would allow us to detect these changes in aqueous venous flow in successful surgeries [3].

Data on subconjunctival and suprachoroidal MIGS devices are currently inadequate. In this special issue, A. Galal et al. reported one of the first results on the clinical efficacy of the XEN-45 implant. Subconjunctival drainage associated with the implant was able to achieve IOP in the low teens, though only 42% of the eyes achieved ≥20% decrease in IOP without medications at 12 months. New modalities of cycloablation, such as ultrasonic circular cyclocoagulation by high-intensity focused ultrasound (HIFU), in their attempt to “re-brand” as MIGS procedures, claim good safety records and no reports of serious complications such as persistent hypotony or phthsis. Hence, HIFU has been performed on patients with early glaucoma naïve to filtration surgery, though detractors may argue that the resultant scleral architecture remodelling is a concern if subsequent filtration surgery is required.

The studies in this special issue highlight that more MIGS surgeons are venturing beyond the context of mild to moderate POAG. With increased access to these devices globally, the glaucoma surgeon is spoilt for choice and is faced with the exciting prospect of tailoring glaucoma surgery to the needs of each patient. Comparative studies are required to evaluate the relative efficacy of different MIGS devices and to ascertain their cost-effectiveness.
